# Intrinsic electrophysiological properties predict variability in morphology and connectivity among striatal *Parvalbumin*-expressing Pthlh-cells

**DOI:** 10.1038/s41598-020-72588-1

**Published:** 2020-09-24

**Authors:** Carolina Bengtsson Gonzales, Steven Hunt, Ana B. Munoz-Manchado, Chris J. McBain, Jens Hjerling-Leffler

**Affiliations:** 1 Laboratory of Molecular Neurobiology, Department Medical Biochemistry and Biophysics, Karolinska Institutet, 17177 Stockholm, Sweden; 2 Section on Cellular and Synaptic Physiology, Eunice Kennedy-Shriver National Institute of Child Health and Human Development, National Institutes of Health, Bethesda, MD 20892 USA

**Keywords:** Cellular neuroscience, Neuronal physiology, Basal ganglia

## Abstract

Determining the cellular content of the nervous system in terms of cell types and the rules of their connectivity represents a fundamental challenge to the neurosciences. The recent advent of high-throughput techniques, such as single-cell RNA-sequencing has allowed for greater resolution in the identification of cell types and/or states. Although most of the current neuronal classification schemes comprise discrete clusters, several recent studies have suggested that, perhaps especially, within the striatum, neuronal populations exist in continua, with regards to both their molecular and electrophysiological properties. Whether these continua are stable properties, established during development, or if they reflect acute differences in activity-dependent regulation of critical genes is currently unknown. We set out to determine whether gradient-like molecular differences in the recently described *Pthlh*-expressing inhibitory interneuron population, which contains the *Pvalb*-expressing cells, correlate with differences in morphological and connectivity properties. We show that morphology and long-range inputs correlate with a spatially organized molecular and electrophysiological gradient of Pthlh-interneurons, suggesting that the processing of different types of information (by distinct anatomical striatal regions) has different computational requirements.

## Introduction

Understanding the cellular diversity of a tissue is of paramount importance for understanding the interactions that underlie its physiological function. This is especially true for the brain where numerous different types of specialized neurons within each sub-region likely process information in distinct manners. The emergence of high-throughput molecular methods, such as single cell RNA sequencing (scRNAseq) has permitted the generation of large data sets from independent research groups that can be compared quantitively^1–3^. Thus, facilitating a move towards consensus at least for the larger clearly separated cell types or classes. A significant challenge is to tie the identity of molecular defined cell types to the more traditionally studied properties that have been used to classify cells over the last century. Additionally, many molecular properties exist in a continuum within the proposed cell classes. Such molecular gradients have been observed in many brain regions^4–6^ but it seems to be a key feature of cells within the striatum^7–10^.

*Parvalbumin-(Pvalb)* expressing, fast-spiking basket cells across the telencephalon share the same developmental origin and have long been considered a homogenous group, with a canonical circuit function that includes feed-forward inhibition^11–14^. Their unique high-frequency action potential (AP) firing and dense local axonal arborization allow them to exhibit strong somatic inhibition onto target cells, making them crucial for the fine-tuning of circuit output^15–17^. Within the striatum, *Pvalb-*expressing cells have been described as the most abundant GABAergic inhibitory interneuron type and are electrophysiologically distinguishable from their cortical and hippocampal counterparts^8,18^. However, scRNAseq data have shown that striatal *Pvalb*-expressing cells are molecularly clearly distinct from their cortical (and hippocampal) counterparts, and appear instead more closely related to striatal *Tyrosine hydroxylase* (*Th)*- and *Choline acetyltransferase* (*Chat)*-expressing cells^8^.

The same scRNAseq study revealed, perhaps surprisingly, that striatal *Pvalb*-expressing cells, unlike those in other telencephalic structures, do not cluster as a unique molecular class. Instead, different levels of *Pvalb*-expression can be detected within a larger group labeled with the gene *Parathyroid hormone like hormone* (*Pthlh*) previously suggested as a marker for cortical chandelier cells^19^. Furthermore, the gradient-wise difference in *Pvalb* expression in Pthlh-cells covaries with a larger transcriptional program. In addition, in a previous study using a PatchSeq approach we detected a range of electrophysiological properties within the Pthlh population, ranging from Fast-spiking-like (FSL) to fast spiking (FS) cells^8^.

The well-established FS profile of Pvalb-cells is characterized by a unique high-frequency firing pattern with minimal adaptation^20^. These features are enabled by short action AP half-width concomitant with a large and fast afterhyperpolarization (AHP). In addition, by virtue of their relatively hyperpolarized resting membrane potential (RMP) and low input resistance (50–150 MΩ), these neurons require greater depolarization to reach firing threshold, and therefore exhibit a strong circuit inhibition in an “all or none” manner^21^. FSL cells share many of these parameters and until now the only major difference reported is that they possess a slower half width and therefore also possess a lower maximal action potential firing frequency^8^. Interestingly, we demonstrated that the molecular gradient within Pthlh cells correlated to this electrophysiological diversity. For example, *Pvalb* expression levels directly correlated with AP half width, suggesting that FS cells express higher levels of *Pvalb* compared to cells with a FSL profile^8^.

However, both molecular and electrophysiological profiles are often dynamic and can be up- or down-regulated within minutes or hours, suggesting that the continuous differences we detect within the Pthlh population may reflect plastic *states* rather than stable properties akin to cell types. For this reason, we set out to try to understand how these molecular and electrophysiological gradients correlate with more stable cellular properties such as morphology, and connectivity.

As mentioned above, all striatal *Pvalb*-expressing cells possess a basket-cell-like morphology, wrapping their axon around the soma of the target cell enabling strong somatic inhibitory control^21^. In addition to basket-cells, cortical and hippocampal *Pvalb*-expressing interneurons also comprises axon-initial segment targeting “chandelier cells and bistratified cells”^22–24^, which to date have not been described in the striatum. Nevertheless, despite their basket-cell features, some variations in morphologies have been reported within striatal *Pvalb*-expressing cells^25^. In addition, the morphologies of Pthlh cells that express lower levels of *Pvalb* are yet to be described. Hence, the continuous differences in molecular and electrophysiological properties might also be reflected in possible morphological diversity.

Several studies have shown a spatial bias of *Pvalb* expression across the striatum, with higher levels in the ventrolateral parts^8,26,27^. These findings, together with the correlation between fast-spiking properties and *Pvalb*-expression, predicts a spatial gradient in electrophysiological properties of high frequency firing Pthlh-cells. Furthermore, since different regions of the striatum receive varying levels of input from distinct extra-striatal regions^28,29^, the gradient-like molecular and electrophysiological differences across the Pthlh-population may be accompanied by differential input strength and origin of innervation. In this paper, using a combination of anatomical, electrophysiological and optogenetic techniques we establish that the spatial gradient of molecular and electrophysiological properties of the Pthlh-expressing population correlates with a gradient in both morphological properties as well as long-range synaptic connectivity. Since these two cellular features are less likely to be acutely remodelled than intrinsic electrophysiological properties and gene expression, our findings suggest that this cellular heterogeneity reflect stable differences in how information is processed in different parts of the striatum.

## Results

### FS inhibitory interneurons possess a ventrolateral bias similar to Pvalb expression within the dorsal striatum

**Figure 1:**
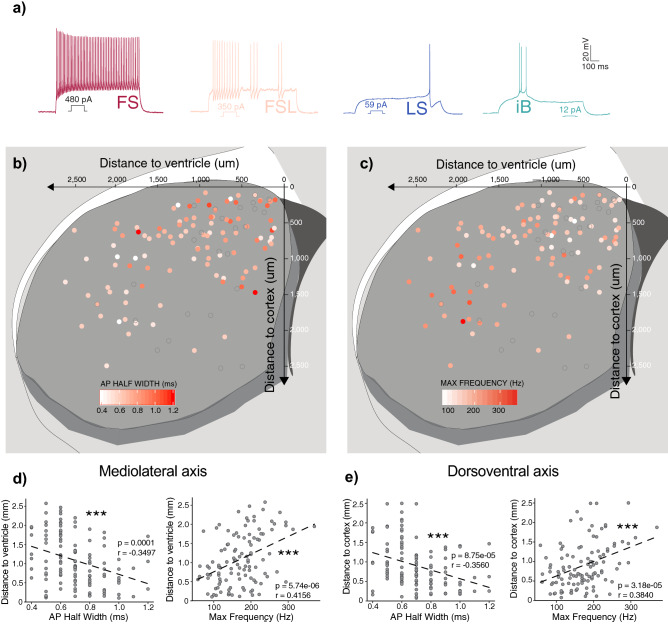
Pthlh cells were selected based on their Fast spiking (FS) or Fast spiking like (FSL) electrophysiological profile, where FS cells show a ventrolateral bias similar to Pvalb expression. (**a**) Representative traces showing typical electrophysiological profiles of FS cells, FSL cells, Late spiking (LS) and Intrinsic bursting (iB) cells. (**b**) Scatterplot showing the AP Half width (ms) across the dorso-ventral (y-axis) and medio-lateral axis (x-axis). (**c**) Scatterplot showing the Max frequency (Hz) across the dorso-ventral (y-axis) and medio-lateral axis (x-axis). (**d**) AP Half width and Max frequency correlated to distance to ventricle (mediolateral axis). (**e**) AP Half width and Max frequency correlated to distance to cortex (dorsoventral axis) (n = 136).

To target the striatal Pthlh population for whole-cell patch clamp recordings, 5HT3a^EGFP^, Pv^cre:TdT^ and Pv^TdT^ mouse lines were used. Together these mice label a variety of striatal overlapping interneuron populations^8^. Based on our previous work we can distinguish these distinct molecular groups solely based on their intrinsic properties^8^. We performed whole-cell patch-clamp recordings from 279 interneurons from ex vivo striatal slices. To selectively investigate the Pthlh population, we included only FS and FSL cells in the analysis. Cells exhibiting other electrophysiological phenotypes, such as intrinsic bursting (IB) or late spiking (LS) phenotypes, which according to our previous studies represent *Th-* and *Melanoma Inhibited activity* (*Mia*)-expressing cells respectively^8^, were excluded (Fig. 1, Supplementary Fig. S1). Despite resembling FSL cells in some electrophysiological paramaters^8^, LS cells are clearly distinct in their AHP shape, AP half with and Latency to spike. When clustered using these parameters LS cells separated clearly from both FS and FSL cells (Supplementary Fig. S1). In addition, they differ both in their morphology (Supplementary Fig. S1) and have distinct molecular profiles^8^, suggesting that there is no gradient-wise cellular differentiation between LS and FSL cells.

During electrophysiological recordings, cells were filled with biocytin included in the intracellular recording solution. Cells were then stained with streptavidin conjugates to allow ad hoc morphological reconstruction and identification of their precise anatomical location. We did not observe any obvious clustering of the electrophysiological data, instead the properties seemed to differ across the Pthlh population in a gradient-wise manner, with the main difference being the frequency of AP firing, which we have previously shown correlates with continuous differences in their molecular profile^8^. In order to introduce as little preconceptions as possible into our analysis we consequently analysed the data as one continuous cell group rather than force a split. We observed a spatial gradient of intrinsic properties along both the ventrolateral and dorsomedial axes. An increase in maximum firing frequency (Hz) and a shorter AP half width (ms) were significantly correlated with medio-lateral axis and dorso-ventral axis (Fig. 1). This spatial distribution of FS-properties within Pthlh cells is reminiscent of our previously reported ventrolateral bias of *Pvalb* expression across the striatum. And as higher levels of *Pvalb* within the Pthlh population correlate with a higher firing frequency^8^, these findings support an hypothesis that the molecular diversity observed within this population also correlates with a spatial gradient of electrophysiological properties.

### FS-properties positively correlate with a more extensive axonal and dendritic arborization

**Figure 2:**
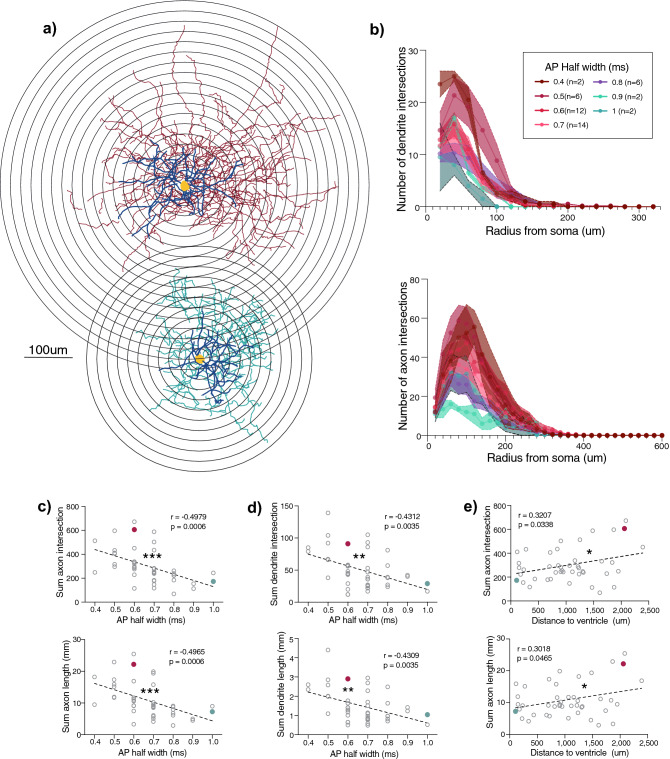
FS cells show more extensive axonal and dendritic arborization, correlating significantly with AP half width and mediolateral axis. (**a**) Representative reconstructions of FS cells in red (upper) and FSL cell in green (lower) and superimposed concentric circles used for Sholl analysis, with a radius interval of 20 µm from the soma. (**b**) (upper) Scholl analysis of dendritic arborization, depicting the number of dendrite intersections (Lower) Scholl analysis of axonal arborization, depicting the number of axon intersections. Both at the same intervals as (**a**), cells were grouped based on AP Half width. (**c**) Correlations of AP half width with sum of axon intersections (upper) and axon length (lower) (n = 44). (**d**) Correlations of AP half width with sum of dendrite intersections (upper) and dendrite length (lower) (n = 44). (**e**) Correlations of distance to ventricle (mediolateral axis) with sum of axon intersections (upper) and axon length (lower) (n = 44).

Next, we wanted to investigate the morphological diversity within the Pthlh population to determine whether the gradient-like differences in electrophysiological profiles, anatomical location, and *Pvalb* expression, were reflected in their morphological diversity. Anatomical reconstruction and morphometric analysis revealed gradient-like differences in both axonal and dendritic arborization that correlated with AP-half width (Fig. 2). In addition, Scholl analysis showed that these differences in cell complexity with distinct AP half was determined mainly within the first 200 µm from the soma for axons and 100 µm from the soma for dendrites (Fig. 2), suggesting that FS cells with higher *Pvalb* expression had more extensive axonal and dendritic arborization than FSL cells with lower *Pvalb* expression. Furthermore, and in concordance with the spatial bias of *Pvalb* expression, we detected differences in morphologies across the mediolateral axis. More specifically, Pthlh cells exhibited a higher complexity of axonal arborization in the lateral striatum compared to the medial part (Fig. 2, Supplementary Fig. S2). No significant correlations were detected between axonal or dendritic arborization along the dorso-ventral axis (Supplementary Fig. S3).

### Forced 2-way split of Pthlh cells based on electrophysiological parameters known to correlate with Pvalb expression

**Figure 3:**
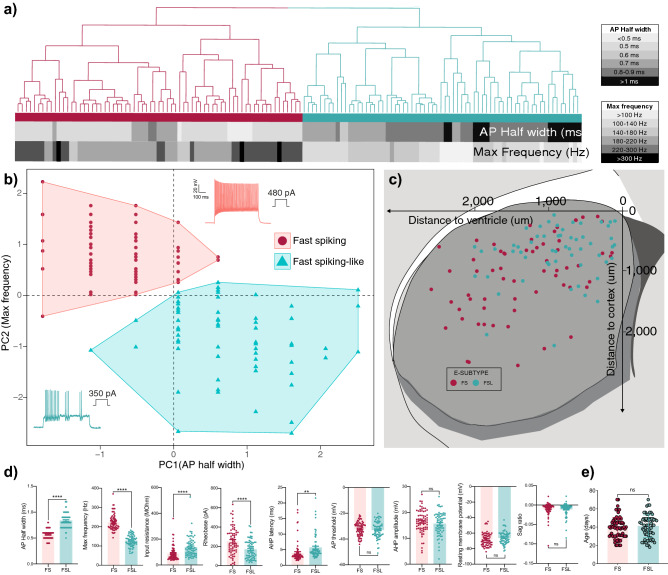
Clustering of Pthlh cells into FS and FSL cells based on selected electrophysiological parameters, known to correlate to Pvalb expression. (**a**) Hierarchical clustering based on Euclidean distance of striatal FS cells (n = 69) and FSL cells (n = 67). Clustering is based on AP Half width and Max frequency. These parameters are shown below, colored from white to black with increasing values. (**b**) Scatterplot showing same clusters derived from the hierarchical clustering in (**a**), with normalized values of Max frequency on the Y axis and AP Half width on X axis. (**c**) Scatterplot showing the distribution of FS (red) and FSL (green) cells across the dorso-ventral (y-axis) and medio-lateral axis (x-axis). (**d**) Differences in electrophysiological parameters between striatal FS (n = 69) and FSL (n = 67) cells. (**e**) Dot plot showing difference in age of FS cells (n = 69) and FSL cells (n = 67). Significance was tested using unpaired t-test. Error bars represent mean ± SEM.

To facilitate our analysis on connectivity we wanted to establish a 2-compartment model in which we could directly compare the mean datasets of FS neurons to FSL neurons. In our previous study, higher AP firing frequency correlated with an increase in *Pvalb* expression within the Pthlh population^8^, and thus this should be an electrophysiological proxy for investigating cells expressing higher *Pvalb* levels to those with lower or none *Pvalb* expression. Hence, we performed k-mean clustering of our dataset using only AP half width and max frequency as parameters, to force-split the recorded cells into two groups, FS and FSL cells (Fig. 3a,b). In accordance with previous results, these populations showed a clear spatial bias, with FSL and FS cells being more prevalent in the dorsomedial and ventrolateral striatum respectively (Fig. 3c). As many electrophysiological parameters are co-dependent, despite only clustering based on AP half width and Max frequency, several additional parameters differed across these two groups. Input resistance (MΩ) and AHP latency (ms) were significantly lower, whereas rheobase (pA) was significantly higher in FS cells in comparison to FSL cells (Fig. 3d). We saw no difference in age between FS (38.8 ± 1.4 days) and FSL cells (41.0 ± 1.5 days) (Fig. 3e). Further supporting our previous finding that FSL and FS cells are not developmental stages of the same cell type^8^.

### FS and FSL cells receive distinct levels of input from motor cortex and thalamus

**Figure 4:**
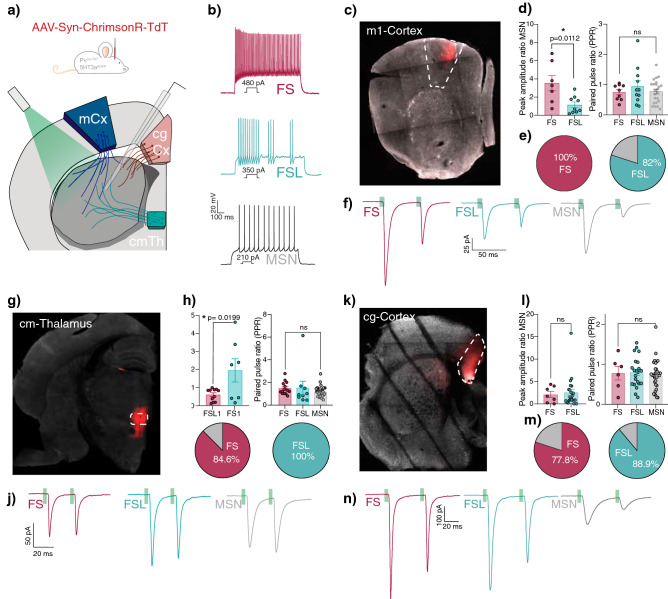
FS and FSL cells receive distinct input from different cortical and thalamic sources. (**a**) Schematic of experimental procedure, including recording of long-range input within the striatum upon injection of AAV-Syn-Chrimson-TdT in 5HT3aEGFP and Pvcre in either motor-cortex (blue), centromedian thalamus (green) and cingulate cortex (pink). (**b**) Representative traces showing typical electrophysiological profiles of FS cells, FSL cells and MSNs. (**c**) Representative pictures of motor cortex injection site. (**d**) (left) Differences in peak amplitude ratio to MSN of motor cortex input onto FS (n = 7) and FSL cells (n = 9). (right) Paired pulse ratio of FS (n = 9), FSL (n = 10) and MSN (n  = 23) when receiving input from motor cortex. (**e**) Pie chart showing connection probability of motor cortex input onto FS (red) (n = 7/7) or FSL (green) (n = 9/11) if input is detected in nearby MSN. (**f**) Example traces showing motor cortex input onto FS cells (red), FSL cells(green) and MSNs (grey) upon paired pulse optogenetic stimulation (20 Hz train of 2 ms light stimulation). (**g**) Representative pictures of centromedian thalamus injection site. (**h**) (left) Differences in peak amplitude ratio to MSN of thalamic input onto FS(n = 11) and FSL (n = 7) cells. (right) Paired pulse ratio of FS (n = 15), FSL (n = 9) and MSN (n = 34) when receiving input from cm Thalamus. (**i**) Pie chart showing connection probability of thalamic input onto FS (red) (n = 11/13) or FSL (green) (n = 9/9) if input is detected in nearby MSN. (**j**) Example traces showing thalamic input onto FS cells (red), FSL cells (green) and MSNs (grey) upon paired pulse optogenetic stimulation (20 Hz train of 2 ms light stimulation). (**k**) Representative pictures of cingulate cortex injection site. (**l**) (left) Differences in peak amplitude ratio to MSN of cingulate cortex input onto FS(n = 7) and FSL cells (n = 23). (right) Paired pulse ratio of FS (n = 6), FSL (n = 22) and MSN (n = 31) when receiving input from cingulate cortex. (**m**) Pie chart showing connection probability of cingulate cortex input onto FS (red) (n = 7/9) or FSL (green) (n = 22/25) if input is detected in nearby MSN. (**n**) Example traces showing cingulate cortex input onto FS cells (red), FSL cells (green) and MSNs (grey) upon paired pulse optogenetic stimulation (20 Hz train of 2 ms light stimulation).

The striatum receives excitatory glutamatergic input from distinct cortical and thalamic areas^28^, with a great extent of locational bias and a varying degree of cellular specificity^26,29–31^. Consideration of this spatial distribution of cortical and thalamic inputs we hypothesized that the varying intrinsic properties within the Pthlh population might correlate with innervation from different sources of long-range inputs. To investigate synaptic differences and preferences in input onto FL and FSL cells, we used an optogenetic approach. We targeted primary motor cortex, centromedian thalamus and cingulate cortex inputs using AAV-syn-chrimson-TdT injections (Fig. 4). The strength and probability of input onto FS and FSL cells from these distinct regions were then detected upon light-induced activation of TdT + fibers in the striatum.

The glutamatergic inputs from different cortical and thalamic sources onto MSNs have been extensively studied. However, anatomical tracing studies show inconclusive results when attempting to quantifying cortical and thalamic inputs onto distinct MSN populations^32,33^. However, in vivo and ex vivo electrophysiological studies support the fact that there is little difference between thalamic and cortical input amplitudes onto indirect and direct MSNs as long as the input arises from the ipsilateral side^34–36^. Hence, in order to avoid biases in the magnitude of input based on variability in virus amount, distance to the injection site and incubation time across experiments, the evoked synaptic amplitudes were normalized to glutamatergic inputs detected in nearby MSNs^37,38^. In addition, only the injected hemisphere was collected to avoid any spurious activation of contralateral inputs. The magnitude of input was then quantified using the “peak amplitude ratio”, calculated by dividing the light evoked input amplitude (pA) recorded in the interneurons with the amplitude of input (pA) recorded in an adjacent MSN. The connection probability, on the other hand, was calculated as the percentage of interneurons in which a response was detected when we also observed a synaptic current in the adjacent MSN.

**Figure 5:**
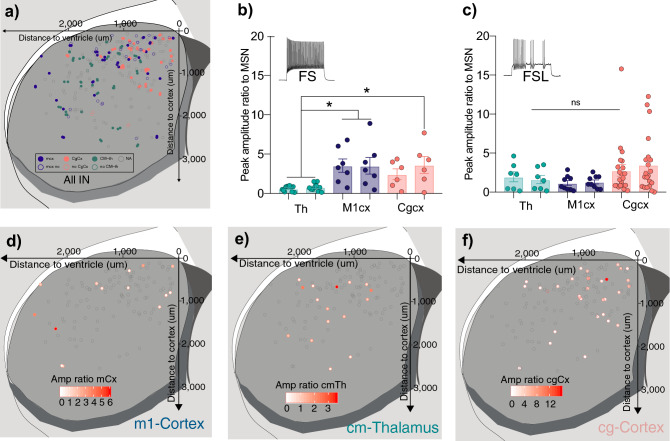
Distinct cortical and thalamic inputs preferentially target different parts of the striatum. (**a**) Scatterplot showing the distribution recorded cells across the dorso-ventral (y-axis) and medio-lateral axis (x-axis). Colored based on input area (orange = cg cortex, blue = m1 cortex, green = CM-th). (**b**) Differences in input amplitudes (peak amplitude ratio to MSN) onto FS cells from CM Thalamus (green) M1 cortex (blue), and Cg cortex (orange). (**c**) Differences in input amplitudes (peak amplitude ratio to MSN) onto FSL cells from CM Thalamus (green) M1 cortex (blue), and Cg cortex (orange). (**d**) Scatterplot showing the distribution recorded cells across the dorso-ventral (y-axis) and medio-lateral axis (x-axis). Colored from white to red with increasing input from motor cortex (peak amplitude ratio of MSN). (**e**) Scatterplot showing the distribution recorded cells across the dorso-ventral (y-axis) and medio-lateral axis (x-axis). Colored from white to red with increasing thalamic input (peak amplitude ratio of MSN). (**f**) Scatterplot showing the distribution recorded cells across the dorso-ventral (y-axis) and medio-lateral axis (x-axis). Colored from white to red with increasing input from cingulate cortex (peak amplitude ratio of MSN).

We could observe that FS cells received greater input, both in terms of amplitude and probability (3.54 ± 0.84, 100%) from motor cortex in comparison to FSL cells (1.16 ± 0.31, 82%) (Fig. 4). However, when investigating thalamic input, FSL-cells received relatively greater input with higher connection probability (1.96 ± 0.864, 100%) when comparing to FS cells (0.62 ± 0.10, 84.5%) (Fig. 4). Cells that responded to motor cortex input were located primarily within the dorsal- as well as the ventrolateral-part of the striatum (Fig. 5). The distribution of thalamus-responding Pthlh cells however seemed to be more concentrated towards the center of the striatum, with somewhat higher responses in the more dorsal parts (Fig. 5, Supplementary Fig. 5). However, we also observed a weak (non-significant) trend suggesting that distinct inputs could perhaps correlate to the electrophysiological and anatomical gradient (Supplementary Fig. 5). This could indicate that the target selectivity of distinct inputs onto the Pthlh population also might occur in a gradient-wise manner.

### Cingulate cortex provides input onto mainly dorsomedial striatum, but equally to FS and FSL cells

In concordance with a previous study^26^, we only observed responses to input from cingulate cortex in the dorso-medial part of the striatum (Fig. 5, Supplementary Fig. 5). Despite the larger prevalence of FSL cells in this region, we did not detect any differences in target cell-preference, as the Pthlh cells, independent of intrinsic firing profile, receive equal inputs in terms of magnitude (FSL: 2.54 ± 0.69, FS: 2.09 ± 0.68) and connection probability (FSL: 88.9%, FS: 77.8%) (Fig. 4, Supplementary Fig. 5).

In general, the cortical input amplitude onto FS cells, both from motor and cingulate cortex was greater than that from thalamus. No such overall difference in cortical and thalamic input was seen within the FSL cells (Fig. 5). However, it is important to bear in mind that MSNs, which are used for the normalizing of the signal, have been shown to receive stronger (higher amplitude) input from cortex than thalamus^35,36,39^. Hence, suggesting that the differences in cortical and thalamic input onto FS cells might be even larger with an alternative normalization method. In addition, cortical inputs from these two areas differ in their target cell-identity nature. While cingulate cortex input lacks target-cell specificity and purely relies on anatomical preference, motor cortex shows greater input onto FS cells in comparison to FSL cells, independent of anatomical location.

## Discussion

In this study, we show that the previously detected gradient-like differences in molecular profile and intrinsic properties within the striatal Pthlh population correlate with differences in morphology and to some extent anatomical location and long-range input. Interestingly, *Pvalb* expression is highly correlated with all parameters mentioned above.

By correlating the morphological profiles and connectivity pattern to AP half width, we could, although indirectly, describe the long-range input as well as morphological profiles of Pthlh cells expressing lower levels of *Pvalb*. Similar to previously described striatal *Pvalb* cells, they exhibit basket-cell-like morphologies, however with gradually less complex axonal and dendritic arborization the lower their *Pvalb* expression. The differences we see in long-range input are partially region-specific, like in the case of cingulate-cortex innervation. However, the input selectivity from motor cortex and thalamus seems to be more cell-type-specific, with FSL cells receiving more of the relatively weaker thalamic inputs and FS cells more of the stronger inputs from the motor cortex. Interestingly, this kind of distinct connectivity pattern within a population exhibiting a continuous molecular gradient has been described in the cortex^6^.

In this study we stimulated the centromedian nucleus (CM). However, other thalamic nuclei also project to the striatum, include the centrolateral (CL) and the parafascicular nucleus (Pf)^35,40^, where the latter has until now been considered more interneuron-specific^41^. Nevertheless, in rodents it is difficult to clearly identify CM and Pf based on solely histological features. For this reason, Pf or CM-Pf complex has often been used to describe both nuclei^42^. Hence, due to the close proximity between these nuclei we cannot exclude a contribution from the PF in our recordings.

In addition to the continuous differences in AP half-width, that we have previously shown to correlate with the gradient-like diversity of molecular profiles^8^, we observed some additional electrophysiological parameters that differed within the Pthlh population. When force-splitting the Pthlh population into FS and FSL cells, it was clear that they did not only differ in the parameters used for clustering (AP half width and Max Frequency). Instead, some additional differences, including higher Input resistance (MΩ) and lower Rheobase (pA) were detected in the FSL population in comparison to the FS population. Hence, suggesting that FSL cells require less current injection to reach firing threshold. This would be in line with FSL being able to respond to the relative weaker thalamic inputs while FS cells are responding more to strong cortical inputs. However, despite being more excitable, FSLs are likely to exhibit a weaker circuit inhibition in comparison to FS cells as their firing frequency once they get excited is lower. Although FS cells have been shown to exhibit feed-forward inhibition within the striatal circuitry^13,43^, the circuit function of FSL cells is still unknown. However, the diversity of excitability and inhibition strength detected within the Pthlh population, suggests that FS and FSL cells most likely also differ in their circuit function.

We do not know specifically when the identity of FS and FSL cells arises. Is it predetermined and guided by diverging molecular codes during development or does it only arise upon circuit integration in distinct parts of the striatum and thereby exposure to distinct types of input during specific developmental windows? Our electrophysiological data in this study, and the molecular data in our previous study^8^, was specifically sampled over pre- and post-adolescent times and we observed no difference in the distribution of these properties of the cells suggesting that this specification occurs before adolescence. As *Pvalb* expression has been shown to be activity-dependent in the cortex^44,45^, it raises the question if the differences we see in any way are activity- or input-dependent. However, due to the more stable nature of some of the readouts used in this study, such as morphology and connectivity we do not think our findings support a model where the *Pvalb* high and low expressing cells within the Pthlh population are short-term activity-dependent states. It is still possible that they are long-term activity-dependent states, influenced by distinct levels of input throughout longer periods of time. Unlike in cortex where several genes have been implicated in cell type-specific activity-dependent chages^44,46^, little is known about interneuron-specific activity-dependent programs in the striatum including the *Pvalb* or *Pthlh*-expressing cells. In order to elucidate this, further studies would be required, including silencing and/or activating striatal Pthlh*-*cells, followed by open-ended molecular readouts such as single-cell RNA-sequencing. Another unresolved question is how to conceptually handle observed continua of properties within cell types or classes; i.e. should we subdivide them or treat these cells as a single cell class? Although this may represent a true feature of the cell system, it may also exist simply from a low sampling methodological problem, and perhaps clarity of the distinct classes would emerge with a larger sample size^47,48^.

Interestingly, a recent snRNA-seq study across several species including mice, non-human primates and humans also detects the Pthlh/Pvalb population as a single molecular cluster^49^. This would suggest that this population is evolutionarily conserved across species. It remains to be determined whether this conservation also holds true for the morphological and electrophysiological profiles of human Pthlh cells. In rat a population of cells expressing *Scgn* that also partly co-express *Pvalb* has also been described^50^. Notably, in rat *Pvalb*-expressing cells the expression of *Scgn* predicts their entrainment to cortical oscillations^51^. Since very few cells in the mouse express *Scgn*^8^ it remains to be investigated if *Scgn*-expression is correlated with a FS or FSL phenotype in other species. Taken together, this study reveals that gradient-like differences are detected across several characterizing features within the striatal Pthlh population, including molecular, electrophysiological and morphological profiles. Despite being correlated, it is unclear if the correlations we observe are causal and how these gradients arise. Nevertheless, FS and FSL cells that are found at different parts of the gradient, and receive different levels of cortical and thalamic input. Thus, indicating that FS and FSL cells are specialized to process distinct types of information. Hence, we hypothesize that the gradient observed might also reflect a continuum of circuit-specific functions.

## Methods

### Animals

Electrophysiological recordings alone and after virus injections were performed in either single or double-positive combinations of the following 3 mouse lines: 5HT3a^EGFP^ (Tg(Htr3a-EGFP)DH30Gsat, GENSAT project, Rockefeller University, NY, USA), Pvalb^cre^ (B6;129P2-Pvalbtm1(cre)Arbr/J, The Jackson Laboratory) crossed onto a Rosa26- tdTomato (Ai14) strain, and Pvalb^TdT^ (C57BL/6-Tg(Pvalb-tdTomato)15Gfng/J, The Jackson Laboratory) in the ages p18–p70. These mice were all on a C57Bl6 background.

For all experiments, both male and female mice were used. Mice were housed on a standard 12 + 12 light/dark cycle with 2–5 mice per cage. All experiments were conducted in accordance with guidelines, permissions and animal protocols approved by the National Institutes of Health’s Institutional Animal Care and Use Committee (IACUC) (ASP#17-045) and Stockholm Norra Djurförsöksetisks Nämd (N18872-2018).

### Electophysiological recordings

For whole-cell patch-clamp recordings mice were anesthetized with isoflourane, prior brain dissection in ice-cold oxygenated cutting solution (in mM): 62.5 NaCl, 2.5 KCl, 1.25 NaH_2_PO_4_, 25 NaHCO_3,_ 10 d-glucose, 100 Sucrose, 7 MgCl_2_, 0.5 CaCl_2_ (pH 7.4).

300 μm thick coronal slices containing striatum were collected using a vibratome (VT-1200S Lecia Microsystems, Bannockburn, IL, USA). Slices were allowed to recover for 30 min at 32 °C and then kept at RT in the same cutting solution described above until recordings when they were continuously perfused with 30–33 °C oxygenated aCSF (in mM): 125 NaCl, 2.5 KCl, 1.25 NaH_2_PO_4_, 25 NaHCO_3,_ 10 D-glucose, 2 MgCl_2_, 2 CaCl_2_ (pH 7.4).

Patch electrodes (borosilicate glass; resistance 3–10 MΩ; Hilgenberg, GmbH) were filled with 20 µl internal solution containing (in mM): 150 K-gluconate, 3 MgCl_2_, 0.5 EGTA, 10 HEPES, 2 MgATP, 0.3 Na_2_GTP. After the addition of 0.3% Biocytin osmolarity reached ~ 290 mOsm and pH 7.34.

Measurements of intrinsic properties were performed as previously described^8^. The maximum firing frequency was determined by applying increasing current injections until action potential failure. At the last current injection prior to failure, the max frequency was calculated from the frequency of the first interevent interval. Persistent barrage firing was induced using continuous depolarizing current injections (1,000–1400 pA, 30 Hz). Postsynaptic glutamatergic input from distinct cortical and thalamic regions were measured upon optogenetic stimulation. Chrimson-TdT positive terminals were activated by paired 2 ms pulses (20 Hz) of green light emitted by Cool LED pE-4000 (Cool LED Ltd, Andover, UK). Recordings were acquired using pClamp 10 and analyzed in Clampfit 10 (Molecular Devices, Sunnyvale, CA). Cells were visualized using a 40 × objective and IR-DIC video microscopy (Zeiss Axioskop 2 FS Plus) and Spot Pursuit camera and SPOT 5.6 software (Diagnostic Instruments, Sterling Heights, MI). Whole-cell recordings were performed using a Multiclamp 700B amplifier and DigiData 1550B (Molecular Devices, Sunnyvale, CA).

### Morphological reconstructions

Upon biocytin filling during whole-cell recording, slices were fixed in 4% paraformaldehyde in phosphate buffer saline solution (PBS) at 4 °C O/N. After permeabilization with 0.3% Triton X-100, slices were incubated in Alexa Fluor 647 or Alexa Fluor 555-conjugated streptavidin (Molecular Probes, Eugene, OR, USA). Slices were re-sectioned using a freezing microtome (Microm, Waltham, MA, USA) to a thickness of 100 μm and subsequently mounted using Mowiol mounting medium (Calbiochem, San Diego, CA, USA). Streptavidin stained cells were imaged using Zeiss LSM710 or LSM800 confocal microscopes (20 × objective). After imaging morphologies were reconstructed and scholl analysis (with 20 μm intersections from the soma) was performed using Neurolucida (MBF Bioscience, Williston VT, USA).

### Stereotaxic injections

For virus injections, mice aged p19–p29 were anesthetized using isofluorane and then fixed to a stereotaxic frame (Stoelting, Wood Dale, IL, USA). After subcutaneous administration of lidocaine, the skull was exposed, and a small hole was drilled open using a dental drill. 0.3 ul AAV virus was then injected at a rate of 0.1 μl/min using a glass-pipette and a microsyringe pump controller (World precision instruments or Stoelting). After injection, the glass capillary was left in the injection site for 10 min before removal. The incision was closed with tissue glue (Vetbond, 3 M) and buprenorphine (0.3 mg/kg s.c.) was administered for analgesic purposes.

For AAV-syn-chrimson-TdT^52^ injections the following stereotaxic coordinates (from bregma) were used for M1 motor cortex (Anterior posterior (AP):0 Mediolateral (ML): ± 1.2 Dorsoventral (DV): − 0.5), cingulate cortex (AP:0.78 ML: ± 0.311 DV:  − 1.393) and centromedian-parafascicular thalamic complex (AP: − 1.155 ML: ± 0.15 DV: − 3.4).

## Supplementary information

Supplementary Figures.
