# Supplementary material for: Intrinsic electrophysiological properties predict variability in morphology and connectivity among striatal Parvalbumin-expressing Pthlh-cells
Source: Sci Rep. 2020 Sep 24;10:15680. doi: 10.1038/s41598-020-72588-1 (PMC7518419; doi:10.1038/s41598-020-72588-1)
Supplement: Supplementary file 1 — Supplementary Figures. [file 41598_2020_72588_MOESM1_ESM.pdf]

Supplementary information

Intrinsic electrophysiological properties predict variability in morphology and connectivity among striatal *Parvalbumin*-expressing Pthlh-cells

Carolina Bengtsson Gonzales[1,2], Steven Hunt[2], Ana B. Munoz-Manchado[1], Chris J. McBain[2], Jens Hjerling-Leffler[1*]

Affiliations:

1. Laboratory of Molecular Neurobiology, Department Medical Biochemistry and Biophysics, Karolinska Institutet, 17177 Stockholm, Sweden
2. Section on Cellular and Synaptic Physiology, *Eunice Kennedy-Shriver* National Institute of Child Health and Human Development, National Institutes of Health, Bethesda, MD 20892, USA.

* Corresponding author (jens.hjerling-leffler@ki.se)

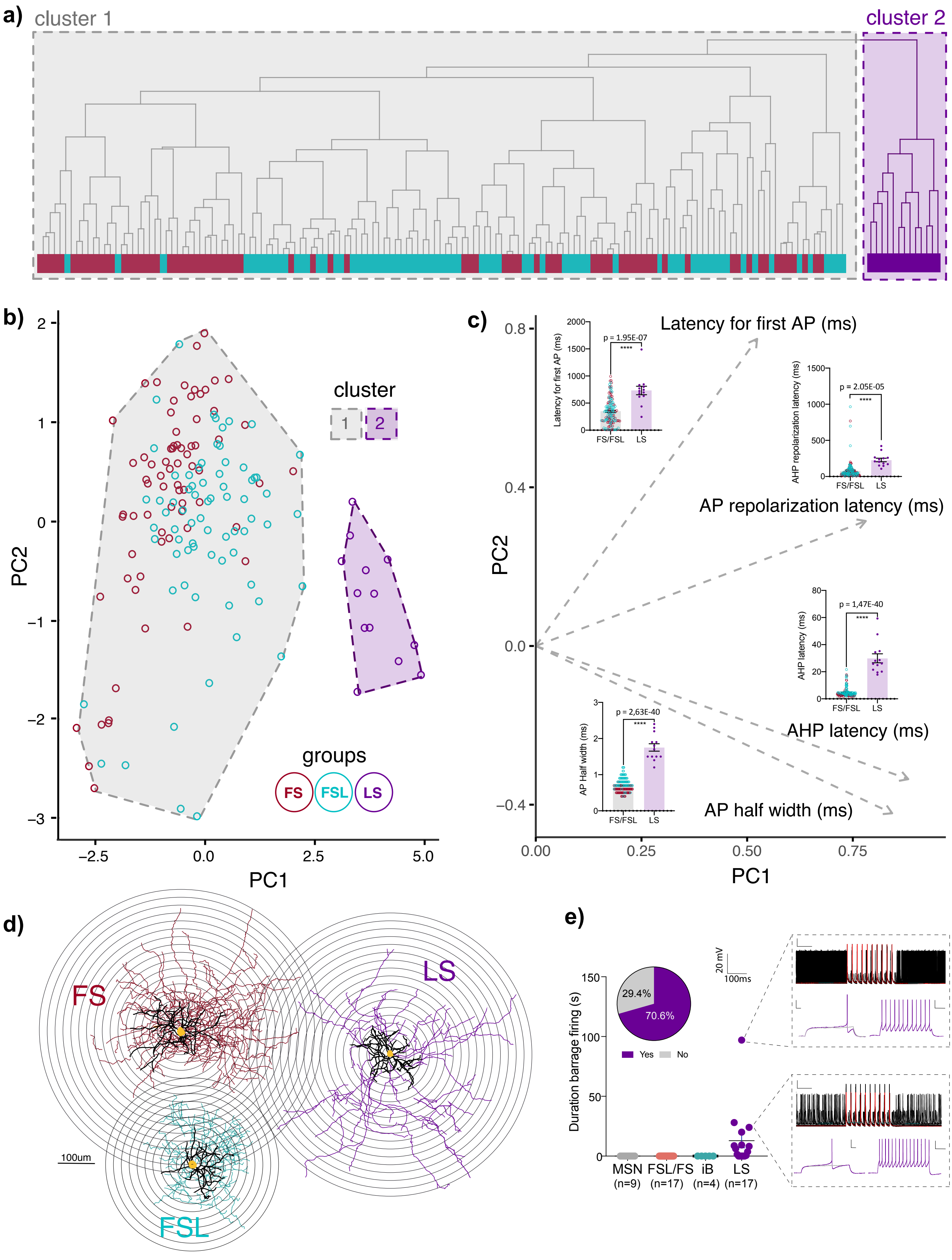

Figure S1. Interneurons population recorded in $5HT3a^{EGFP}$, Pv^{TdT} and Pv^{cre} animals

(a) Hierarchical clustering based on Euclidean distance of striatal FS cells (n=72),FSL cells (n=72) and LS cells (n=13). Clustering is based on AHP latency, AHP repolarization latency, AP half width and Latency to first AP. **(b)** PCA based on the previously mentioned parameters and cells. **(c)** Vector factor map analysis showing the parameters used in the PCA and their contribution (arrow size). For each parameter a dotplot showing the difference between FS/FSL and LS cells. Significance was tested using unpaired t-test. Error bars represent mean ± SEM. **d)** Representative reconstructions of FS cells in red (upper-left), FSL cell in green (lower-left) and LS cell in purple (right). **(e)** Dotplot showing duration of barrage firing across distinct cell types (MSNs, FS/FSL, iB and LS-cells) with two example traces of LS-cell that exhibit barrage firing. Pie chart showing the proportion of LS cells recorded in the striatum that barrage fire.

a)

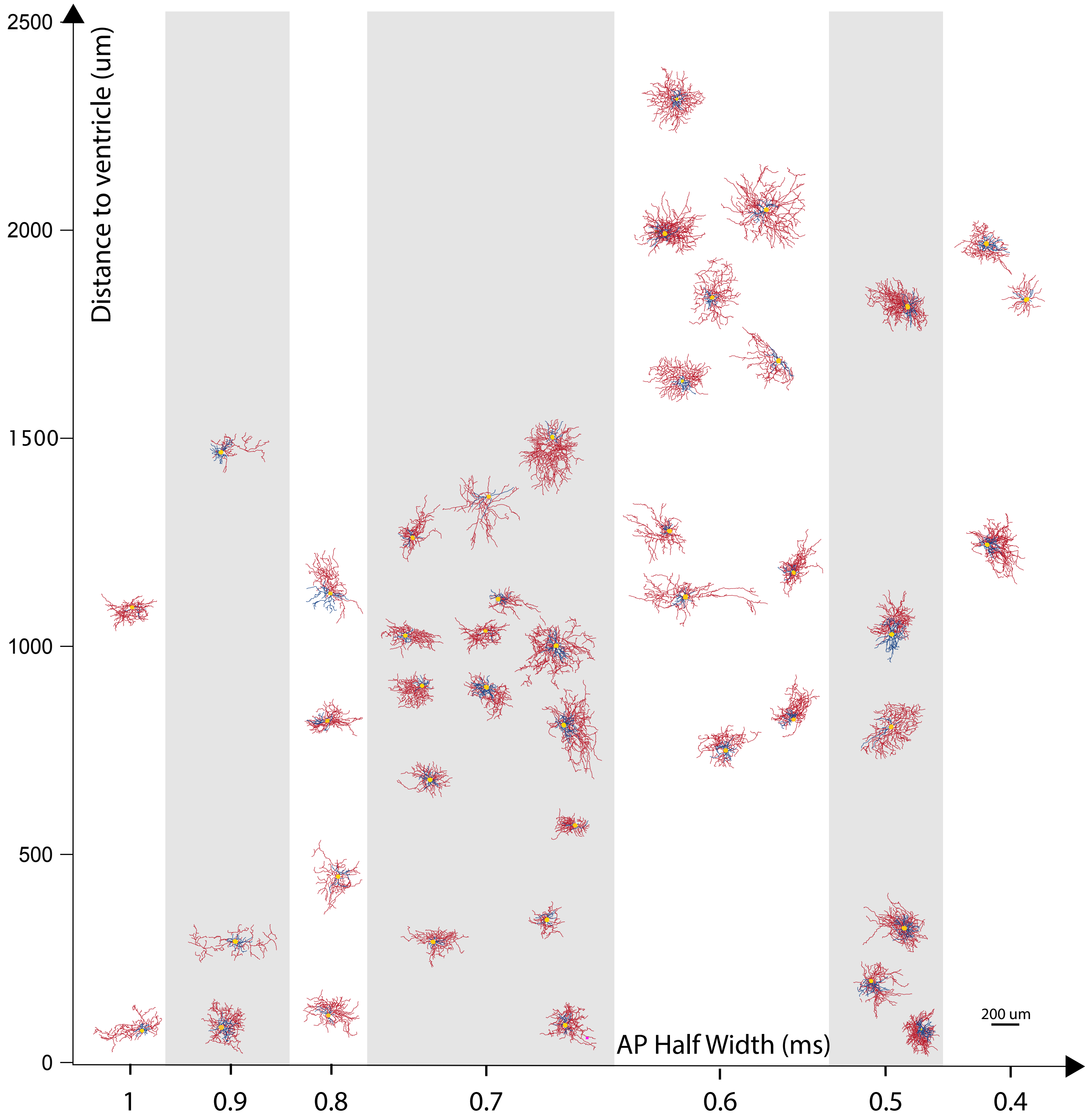

Figure S2. Pthlh cells vary in axonal and dendritic arborization, based on AP half width and location within the mediolateral axis

(a) All reconstructed morphologies of Pthlh cells included in Fig 2 and Fig S3, distributed with increasing levels of AP half width (ms) and distance to ventricle (mediolateral axis)

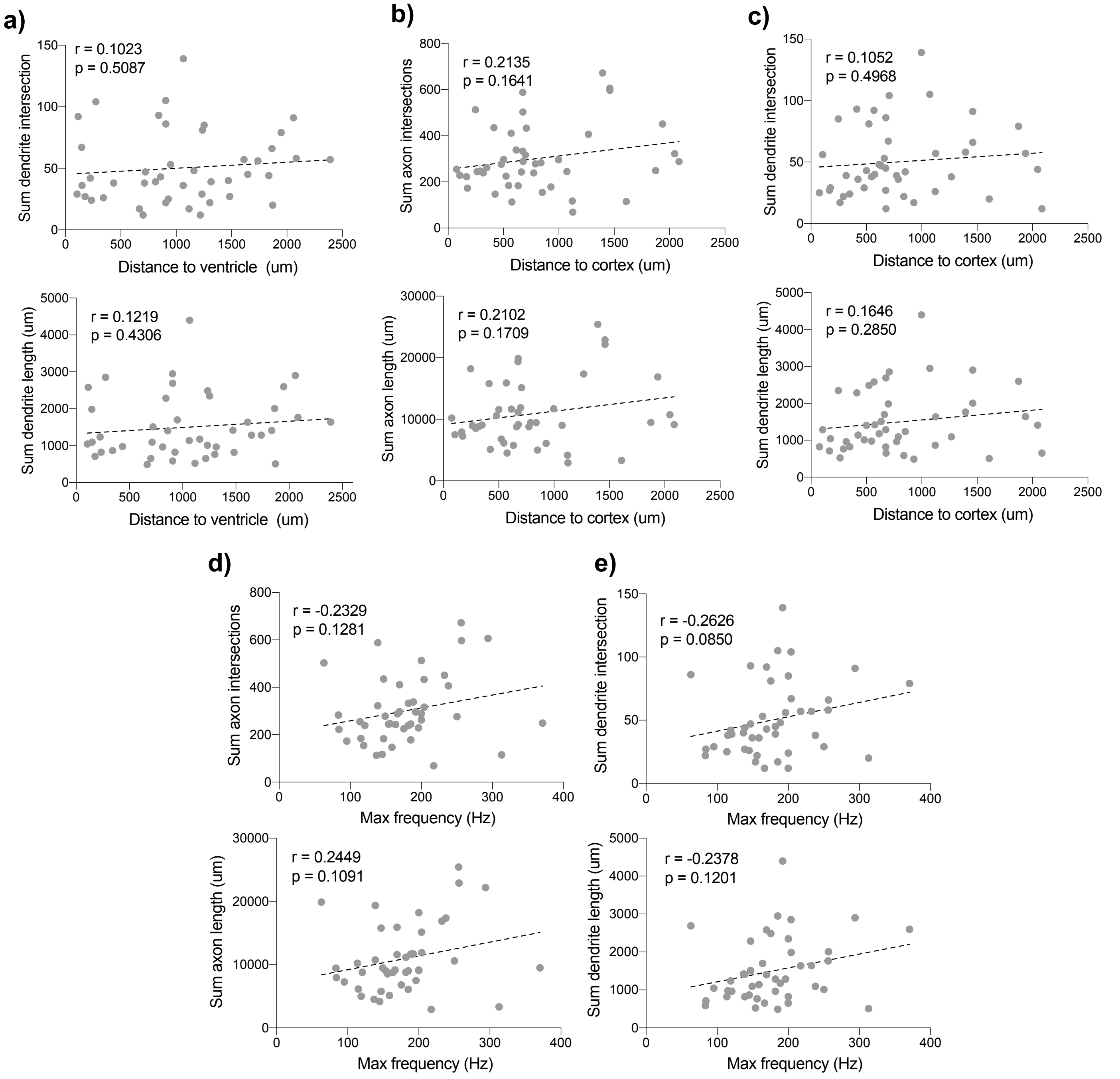

Figure S3. Extent of axonal and dendritic arborization in relation to location within the mediolateral and dorsoventral axis as Max Frequency (Hz)
a) Correlations of distance to ventricle (mediolateral axis) with of dendrite intersections (upper) and dendrite length (lower) (n=44).**b)** Correlations of distance to cortex (dorsoventral axis) with of axon intersections (upper) and axon length (lower) (n=44). **c)** Correlations of d distance to cortex (dorsoventral axis) with of dendrite intersections (upper) and dedrite length (lower) (n=44). **d)** Correlations of Max frequency (Hz) with sum of axon intersections (upper) and axon length (lower) (n=44).**e)** Correlations of Max frequency (Hz) with sum of dendrite intersections (upper) and dendrite length (lower) (n=44).

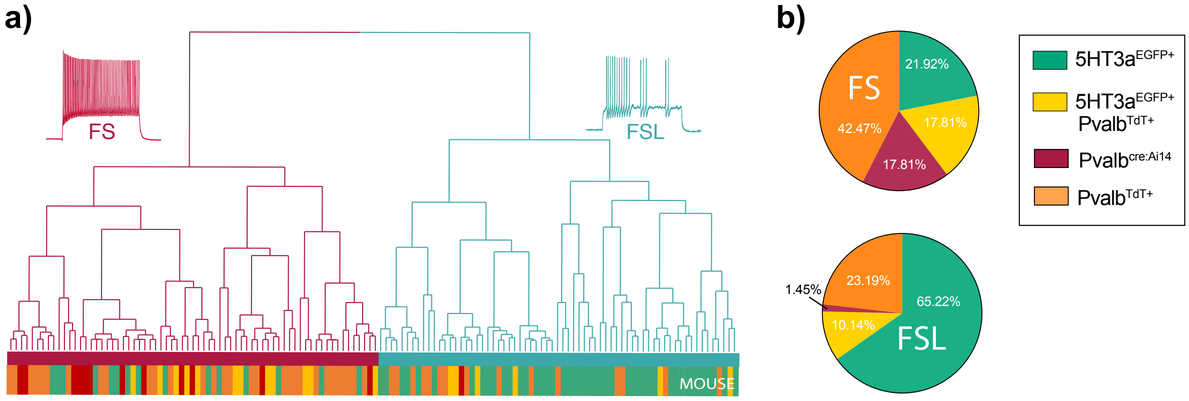

Figure S4. Proportion of FS and FSL cells collected from different transgenic mouse lines (a) Same hierarchical clustering as Fig3A. Mouse genotype in green (5HT3aEGFP), yellow (5HT3aEGFP:PvalbTdT), red (Pvalbcre:Ai14) and orange (PvalbTdT). **(b)** Pie chart showing percentages of the distinct mouse lines where FS cells (n=69) and FSL cells (n=67) were recorded from.

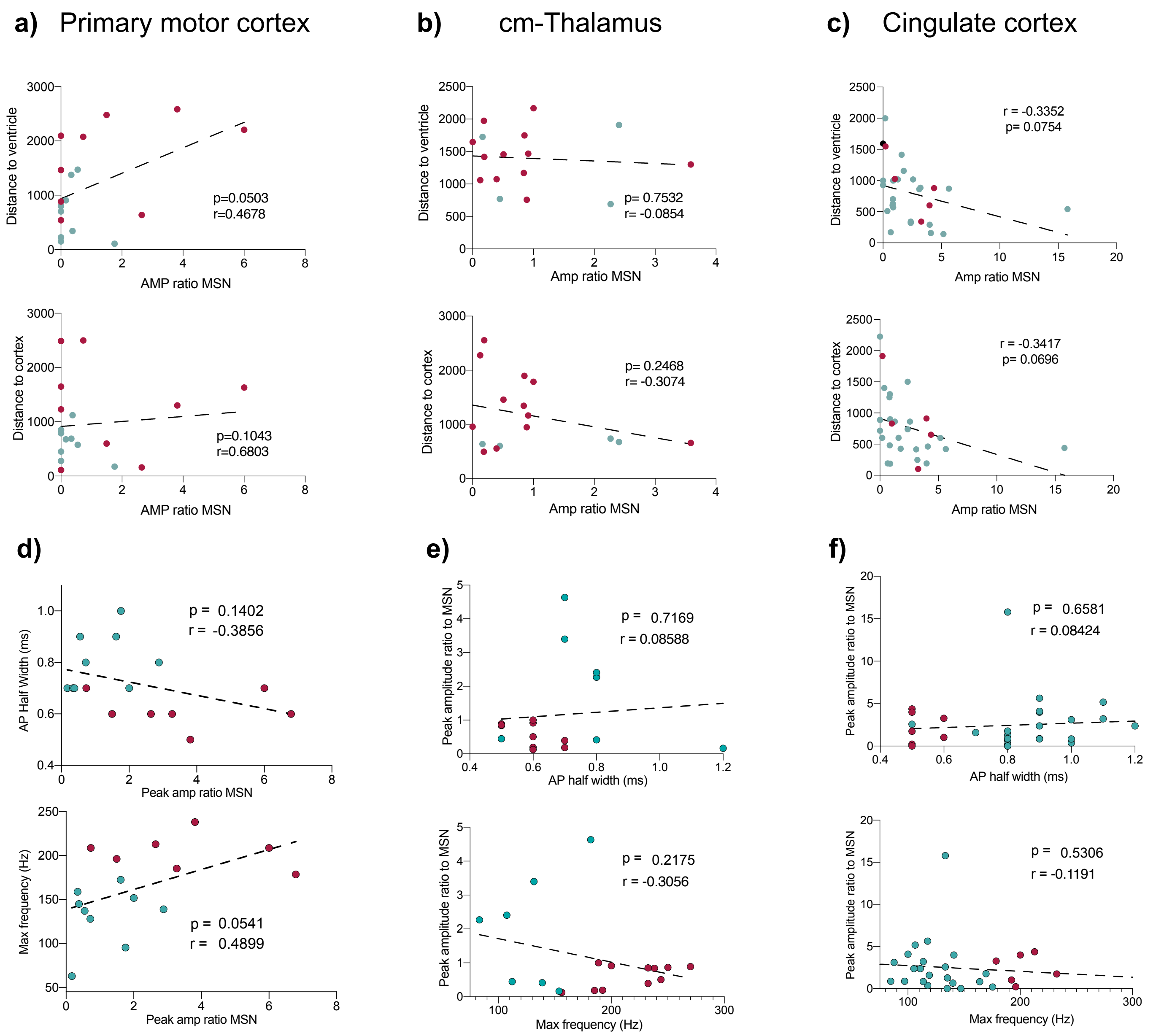

Figure S5. Gradient-like spatial and electropysiological differences in cortical and thalamic inputs onto the Pthlh population

(a) (upper) Correlation of distance to ventricle (mediolateral axis) and (lower) distance to cortex (dorsoventral axis) and Peak Amplitude ratio to MSN of motor cortex input onto FS cells (red dots) and FSL cells (green dots). **(b)** Same as (a) but for thalamic input **(c)** Same as (a) but for cingulate cortex input. **(d)** Peak amp ratio of input from M1 cortex onto FS cells (red dots) and FSL cells (green dots), correlated with AP Half width (upper row) and Max frequency (lower row). **(e)** same as (d) but for CM thalamus. **(f)** same as (d) but for Cg cortex.
